# Supplementary material for: Functional interpretation of single cell similarity maps
Source: Nat Commun. 2019 Sep 26;10:4376. doi: 10.1038/s41467-019-12235-0 (PMC6763499; doi:10.1038/s41467-019-12235-0)
Supplement: Supplementary file 1 — Supplementary Information [file 41467_2019_12235_MOESM1_ESM.pdf]

Functional Interpretation of Single-Cell Similarity Maps

David DeTomaso* Matthew Jones* Meena Subramaniam Tal Ashuach
Chun J. Ye Nir Yosef†

*Authors Contributed Equally
†Correspondence to: niryosef@berkeley.edu

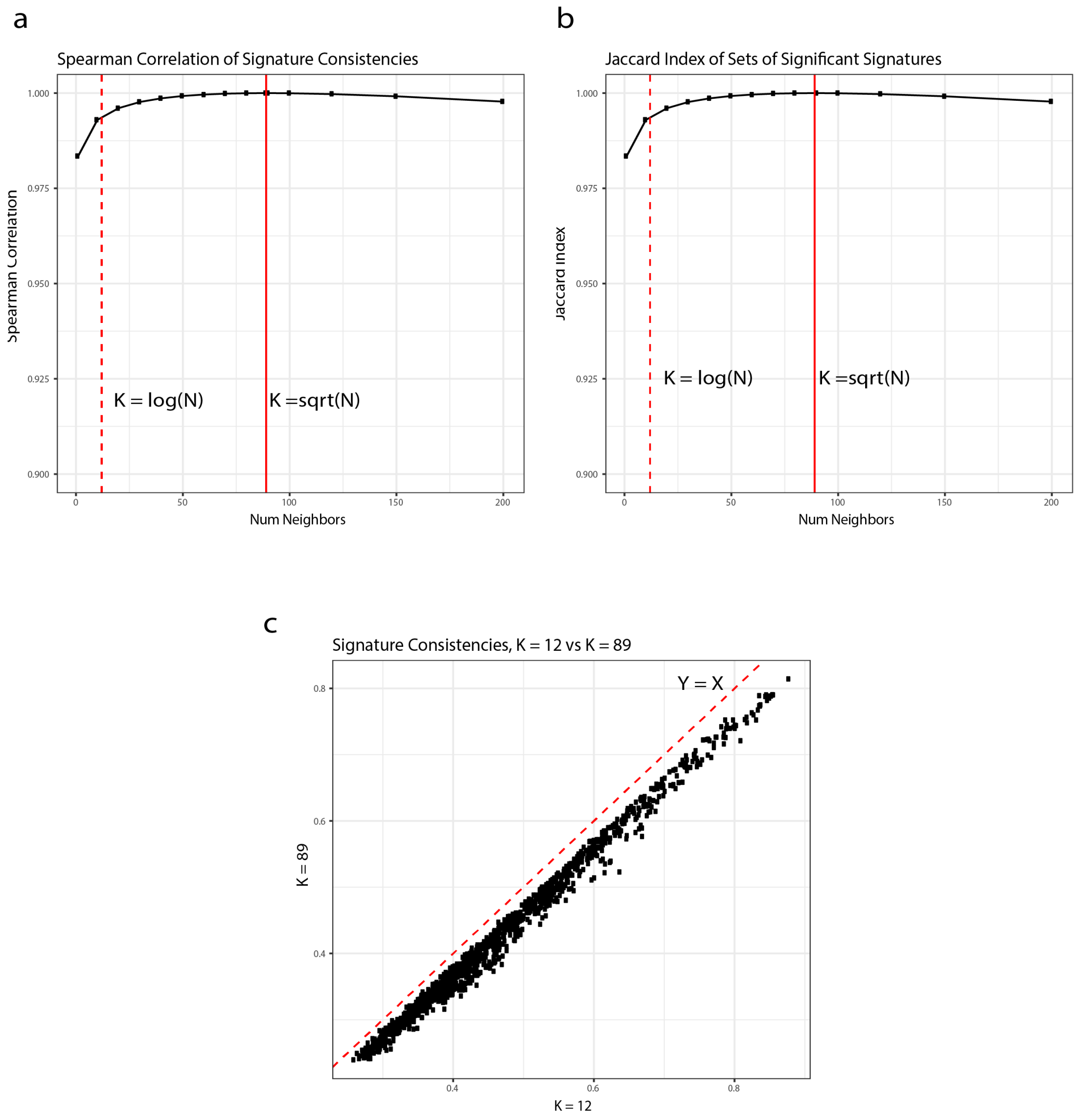

Supplementary Figure 1: **Stability analysis of K, the number of neighbors in the cell-cell similarity graph.** We performed stability analysis by comparing signature consistency scores for the default value of K ($\lceil \text{sqrt}(N) \rceil$) and a range of other K values, from 1 to 200. For this analysis, we used a subset of the AML dataset presented in the paper [1], consisting of 7,780 monocyte and monocyte-like cells. (a) We assessed the rank correlation of the consistency scores of those signatures identified as significant with $K = \lceil \text{sqrt}(N) \rceil = 89$ neighbors as K was changed. Notably, the same signature set were used throughout - namely, those found with an adjusted p-value of less than 0.05 in the default analysis (see Methods). (b) The Jaccard Index is reported for varying K, comparing the set of signatures found to be significant for varying K to the set of signatures found to be significant with $K = 89$ (see Methods). (c) Correlation of signature consistency scores for $K = \log(N) = 12$ and $K = 89$.

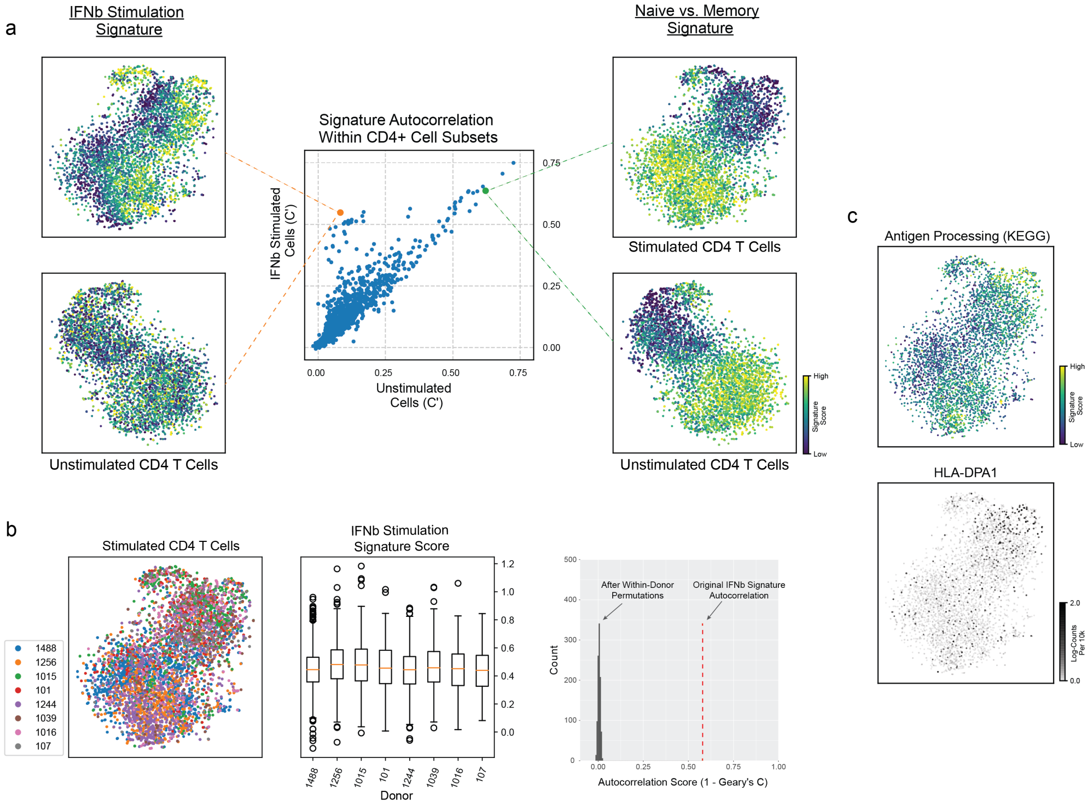

Supplementary Figure 2: **Supplemental figure for Interferon-Stimulated Lupus data.** A) Signature autocorrelation scores (center panel) are compared within the CD4 T cell stimulated vs. unstimulated subsets for a collection of 1057 signatures from MSIGDB[2], KEGG[3], and [4]. Among signatures whose C' coefficients differ greatly between the samples, an Inteferon Beta stimulation signature (from [4]) is clearly distinguished as driving variation within the stimulated cell but not the unstimulated controls. In comparison, a Naive vs. Memory signature (MSIGDB, derived from [5]), has high autocorrelation in both sets of T cells. B) (Left) Stimulated CD4 cells (tSNE) colored by donor ID. (Middle) per-donor distributions of IFNb signature scores (From A). Box-plot elements as follows: center line, median; box limits, lower and upper quartiles; whiskers, 1.5x interquartile range; points, outliers. (Right) Histogram of autocorrelation scores computed under 1000 within-donor permutations of the IFNb signature scores are compared with the observed signature autocorrelation. C) An antigen-processing signature (from KEGG [3]) exhibits a distinct pattern of expression among stimulated CD4 T cells. This signature consists of MHC class 1 and class 2 genes with HLA-DPA1 expression shown as an example.

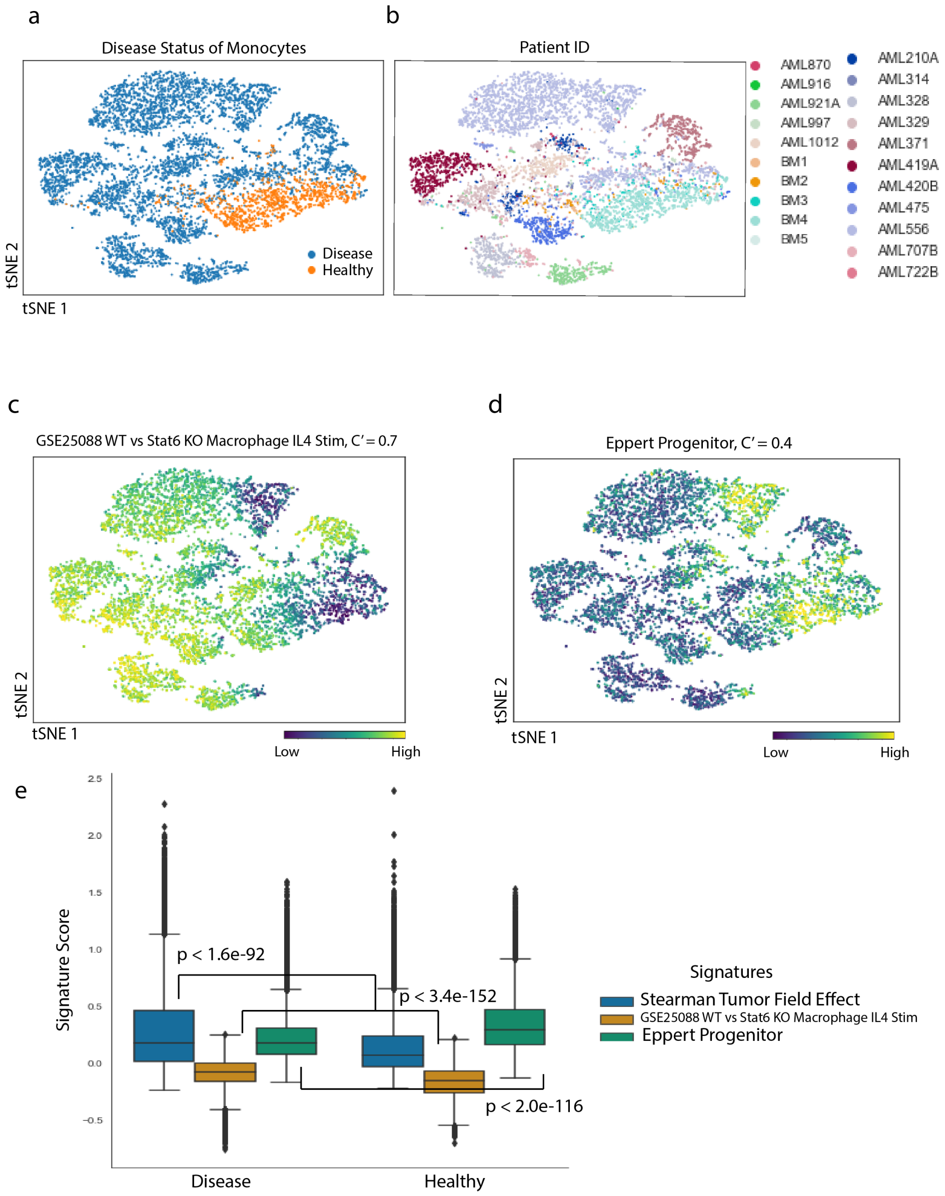

Supplementary Figure 3: **Supplemental figure for AML data.**(a) Disease status of monocytes, indicating whether or not a cell came from an AML patient or healthy bone marrow (Cramer's $V = 0.75, p < 2.7 \times 10^{-3}$). (b) Patient ID, indicating which patient a cell came from ($V = 0.52, p < 2.7 \times 10^{-3}$). (c-d) Signatures highligting the axes of transcriptional variation in the monocyte population: (c) GSE25088 WT vs STAT6 KO Macropihage IL4 Stim ($C' = 0.7, p < 2.7 \times 10^{-3}$) and (d) Eppert Progenitor ($C' = 0.49, p < 2.7 \times 10^{-4}$). (e) Differential signature analysis between disease and healthy patients for Stearman Tumor Field Effect, GSE25088 WT vs Stat6 KO Macrohpage IL4 Stim, and Eppert Progenitor. Significance is calculated using *Vision*'s differential signature test (Methods). Box-plot elements as follows: center line, median; box limits, lower and upper quartiles (25th and 75th); whiskers, 1.5x interquartile range; points, outliers.

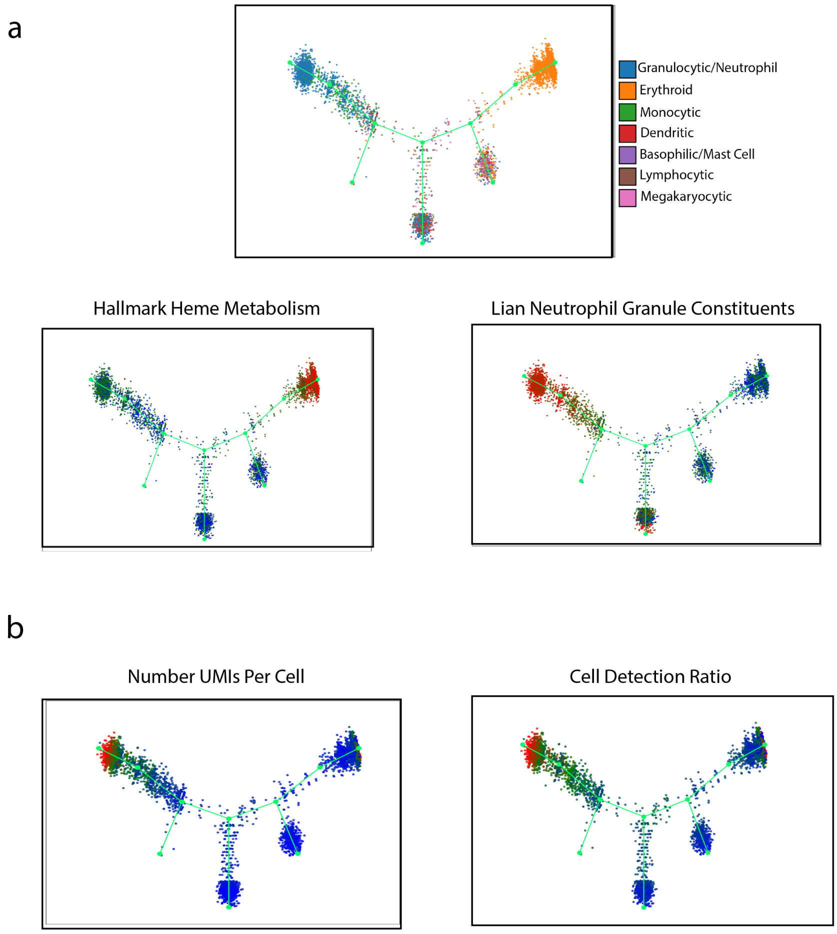

Supplementary Figure 4: **Cell-type specific signatures differentiate branches of an inferred hematopoietic trajectory.** (A) Trajectory representation of cells undergoing hematopoiesis, colored by the cell type's inferred by Tusi et al. [6] in the original study. Hallmark Heme Metabolism, a cell-type signature for the erythrocytic lineage, aligns well with where erythrocytes are found in the trajectory. Lian Neutrophil Granule Constituents, a cell-type signature for the granulocytic lineage, aligns well with where granulocytes are found in trajectory. (B) Cell level meta-data can be used as a data-driven signature; in this case, the number of UMIs and cell detection ratio (CDR; the ratio of detectable genes per cell) are strikingly localized to the granulocyte arm likely due to their increased diameter (16 μm vs $\sim 8\mu$m) compared to other white blood cells and erythrocytes.

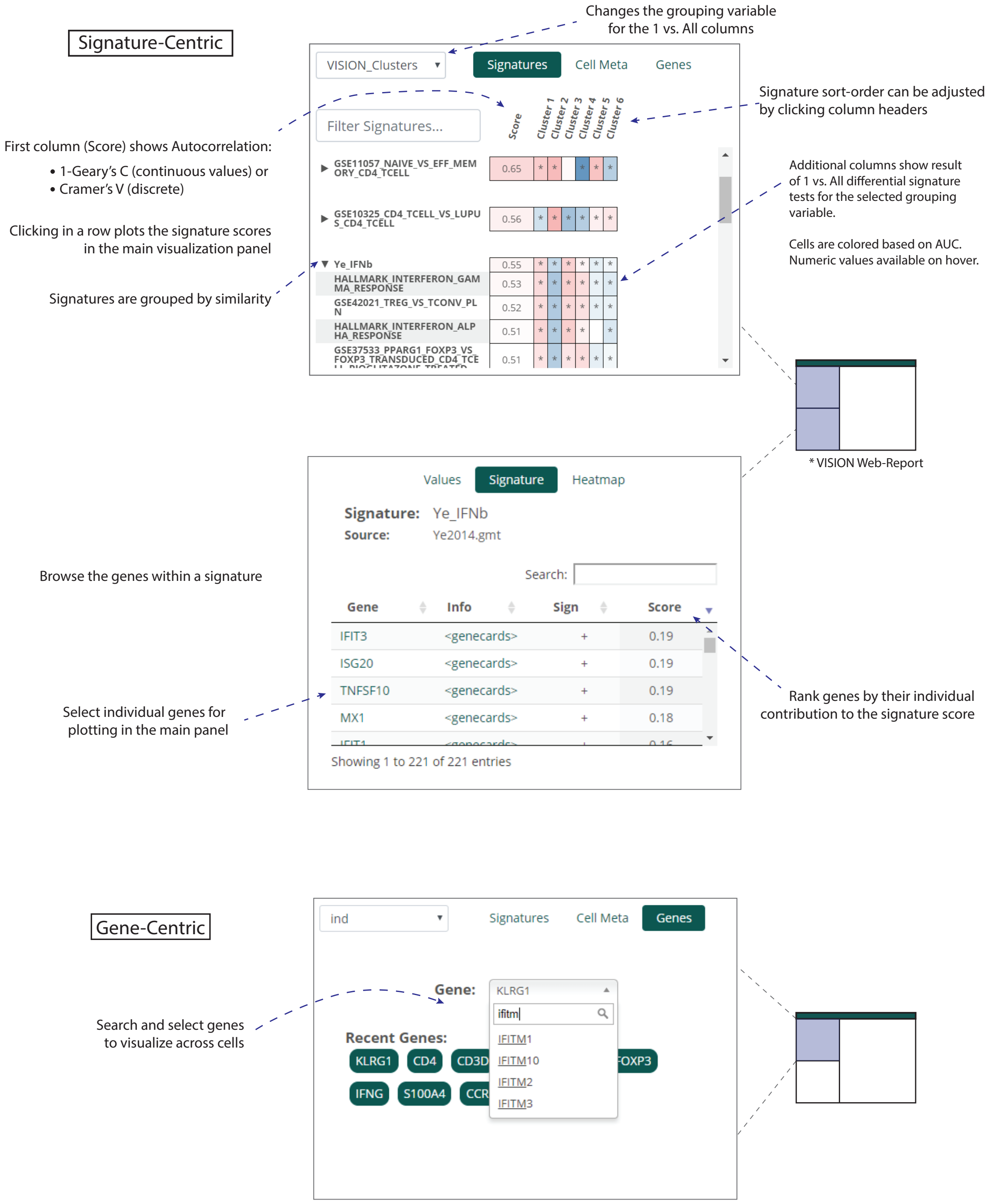

Supplementary Figure 5: **Elements of the output report interface**

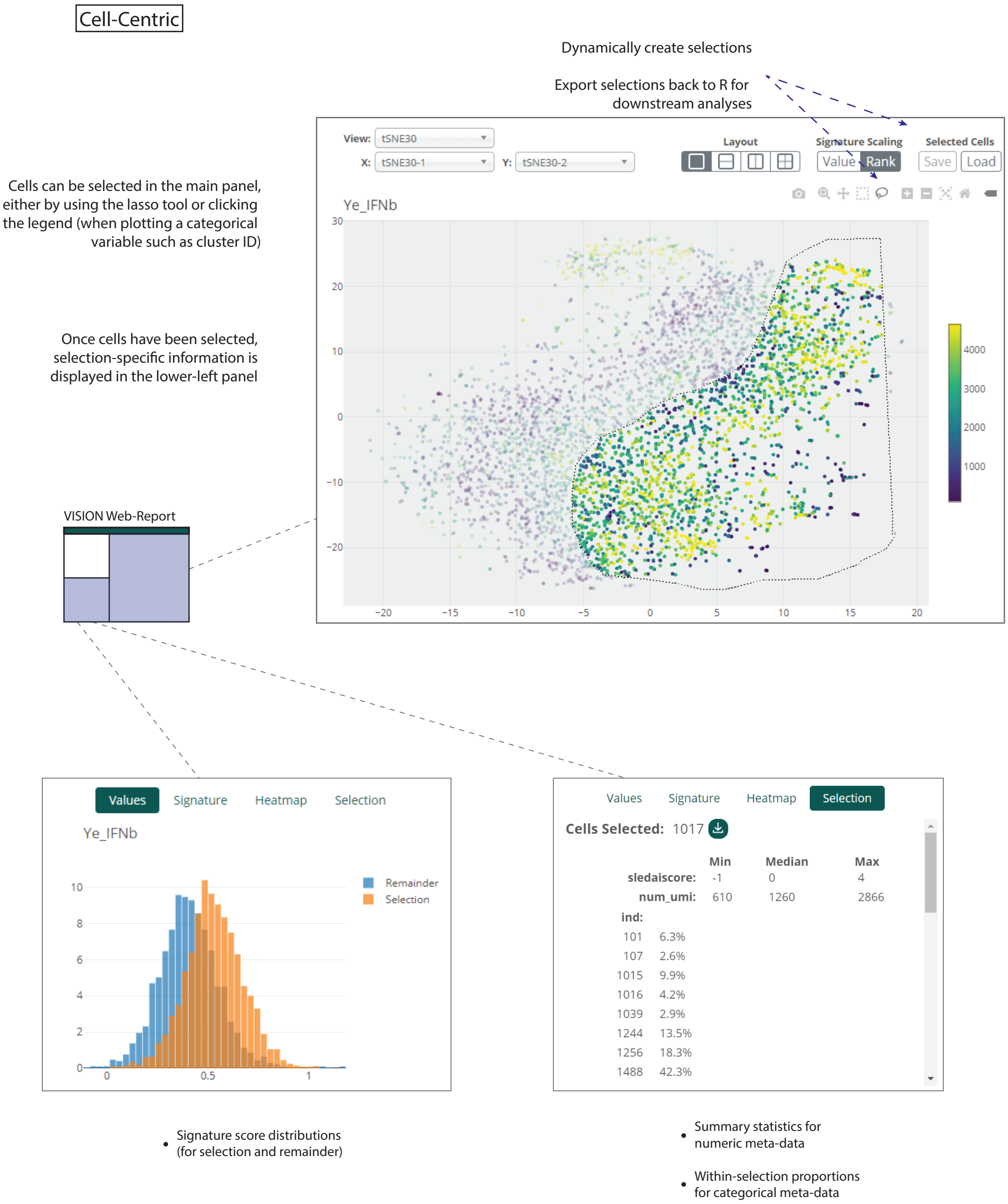

Supplementary Figure 5: **Elements of the output report interface - continued.**

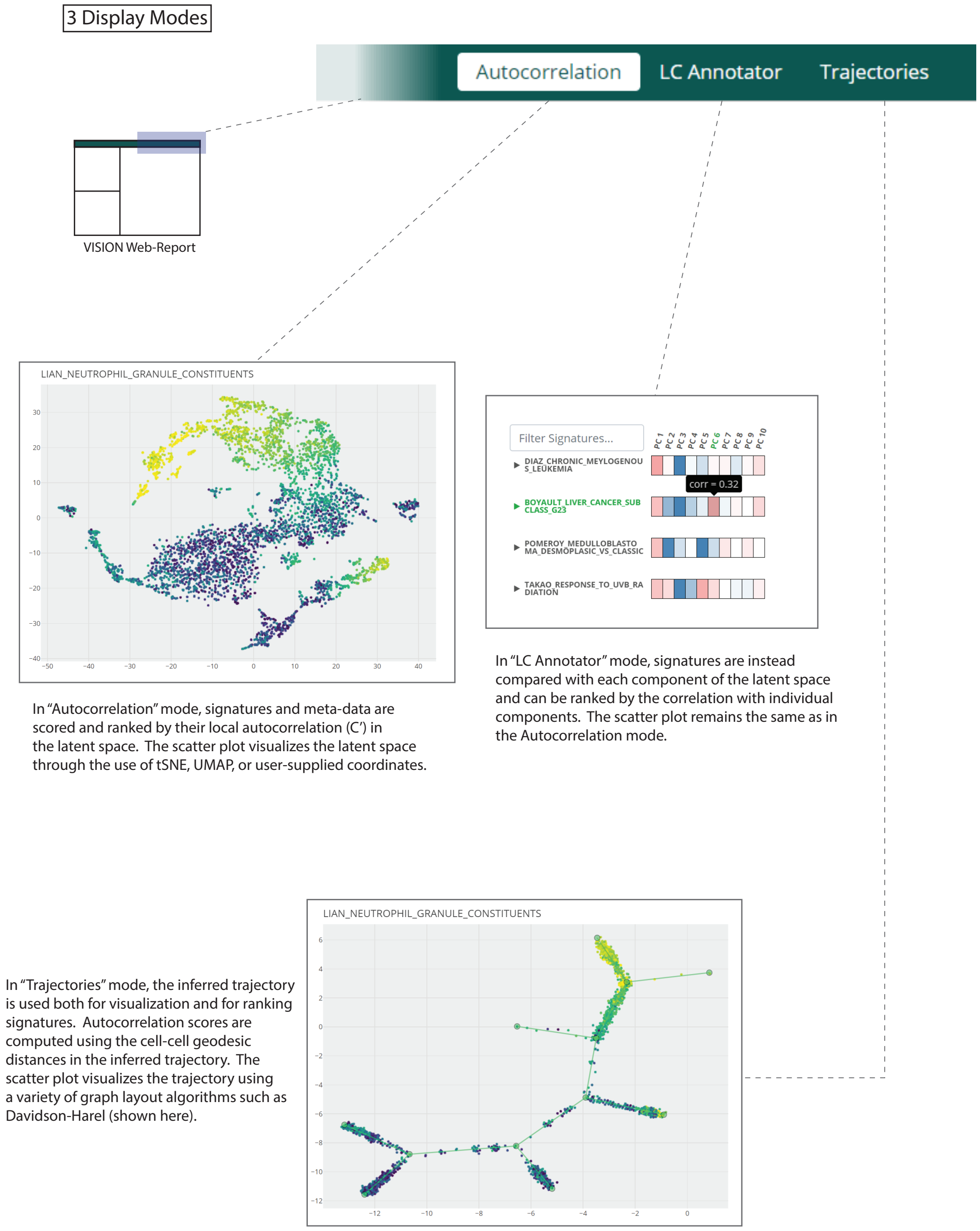

Supplementary Figure 5: **Elements of the output report interface - continued.**

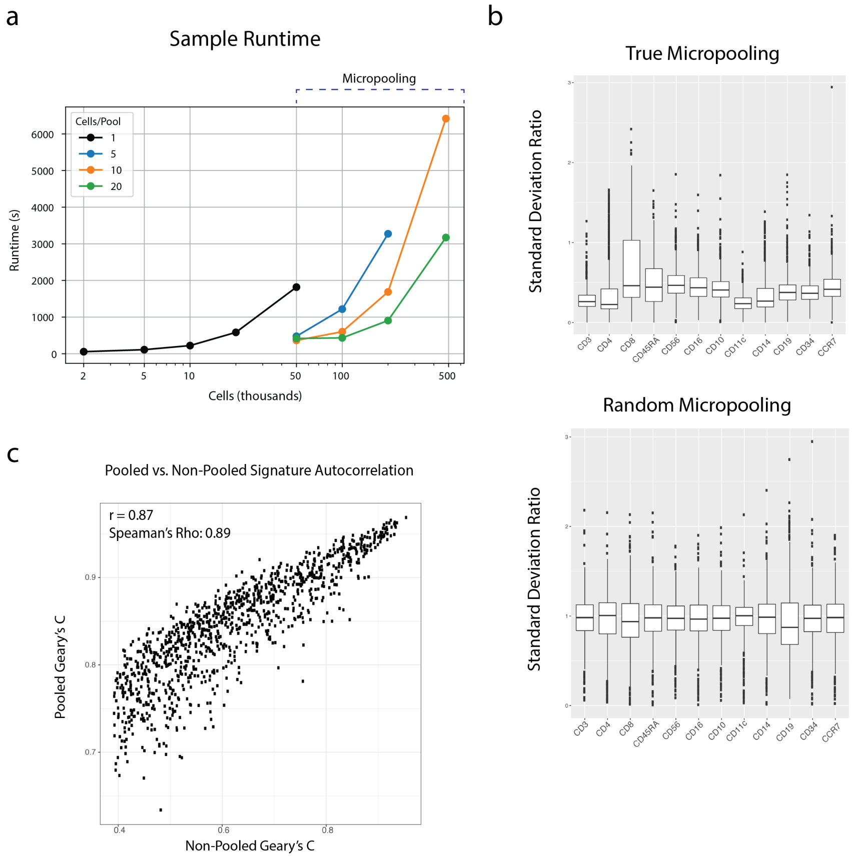

Supplementary Figure 6: **Micro-pooling allows *Vision* analysis to comfortably scale to very large cell counts.** a) Sample runtime for the full pipeline (10 cores). b) To confirm that similar cells were being clustered, we used 9,000 Cord Blood Mononuclear Cells (CBMCs) whose mRNA and protein abundances were profiled simultaneously with the Cite-seq protocol [7]. We find that the cells in each micro-cluster are biologically coherent - specifically, the variation in measured surface protein abundance within micro-clusters is much less than the variation within randomly partitioned micro-clusters. Box-plot elements as follows: center line, median; box limits, lower and upper quartiles (25th and 75th); whiskers, 1.5x interquartile range; points, outliers. c) The signature autocorrelation scores are compared before and after micro-pooling.

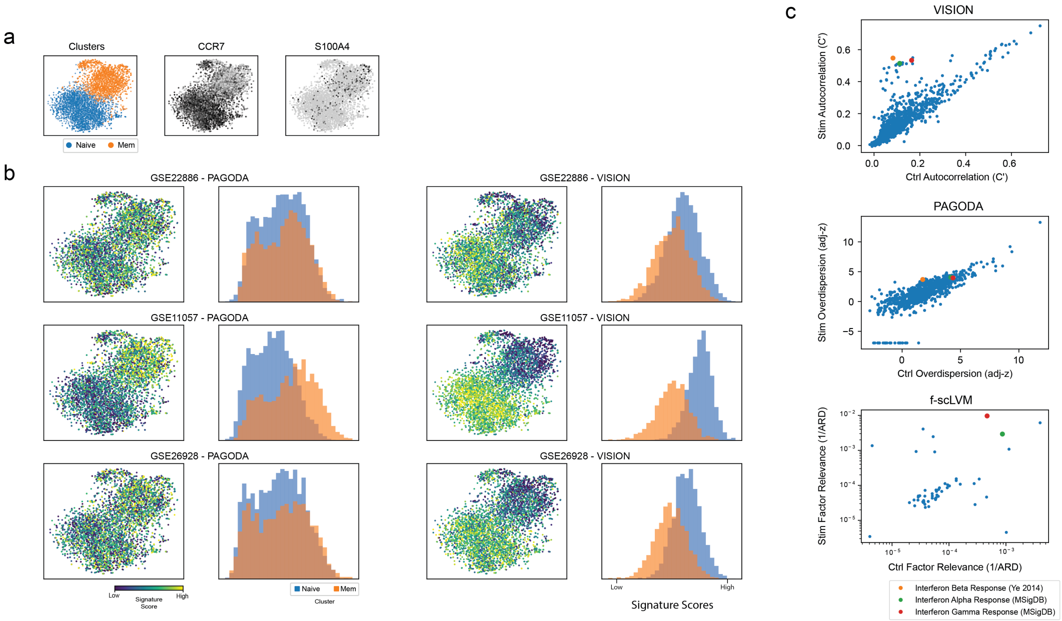

Supplementary Figure 7: **Comparison of signature scores and signature rankings between *Vision* and PAGODA.** A) Stimulated CD4 cells naturally divide into two main clusters, which likely represent naive and memory phenotypes (CCR7 and S100A4 shown). B) Three different naive vs. memory signatures are used (from MSIGDB[2]). For each, the scores computed by *Vision* more clearly distinguish the two clusters than those derived from PAGODA on the same signatures. C) Comparison of the signature rankings produced by *Vision* (local autocorrelation), PAGODA (over-dispersion), f-scLVM (signature importance). The IFNb response signature (from [4] and MSigDB) are more distinguished within the stimulated CD4 cells (when compared with unstimulated) when using the local autocorrelation as a metric.

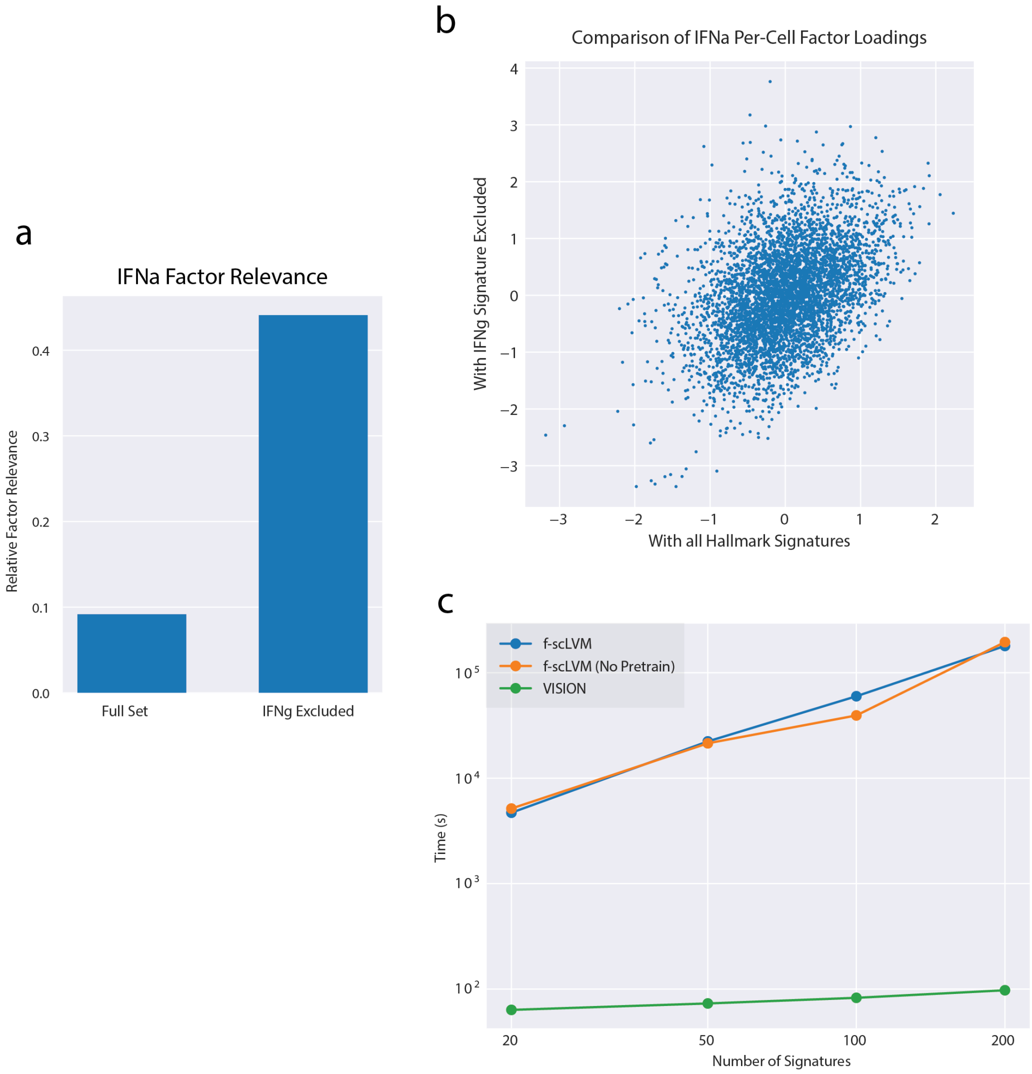

Supplementary Figure 8: **Comparing *Vision* to latent variable models.** f-scLVM, an existing latent-variable model for scRNA-seq data, was run on the interferon beta stimulated CD4 T cells from [8] using the Hallmark signature library from MSigDB[2]. A) The factor relevance of the Interferon Alpha Response signature is compared both when running f-scLVM with the full Hallmark set and when excluding the similar Interferon Gamma Response signature. Values shown are relative to the sum of all signature relevance scores within the run. B) For the same comparison as in (A), the per-cell signature loadings for the Interferon Alpha Response signature are plotted. These results demonstrate that both the factor relevance scores and the individual per-cell signature loadings can be greatly affected by the presence of signatures which capture similar biological signals. C) The runtime of *Vision* and f-scLVM analysis is compared as the number of signatures varies. Results shown are computed on 4773 cells using 28 cores. This demonstrates that f-scLVM is less suitable for analyses using both a large number of cells and a large number of signatures.

Section	Feature	Spring	CCS	Roma	PAGODA	f-scLVM	MAST	Scanpy	Seurat	VISION
Analysis	Evaluation of Pathway Scores			✓	✓	✓		✓	✓	✓
Analysis	Differential Pathway Analysis		✓				✓			✓
Analysis	Confounding of Factor Meta-Data									✓
Analysis	Confounding of Numerical Meta-Data									✓
Analysis	Label-free signature rankings			✓	✓	✓				✓
Analysis	Allows input of Latent Space									✓
Analysis	Allows input of Trajectories									✓
Visualization	Visualize Latent Space							✓	✓	✓
Visualization	Visualize Trajectory	✓						✓		✓
Visualization	Dynamic Web Visualization	✓			✓					✓

Supplementary Table 1: **Feature-wise comparison on *Vision* to existing tools.** *Vision* has several important properties that distinguish it from other software packages for automated annotation and for visualization and exploration of single cell data. First, *Vision* has a comprehensive set of data analysis capabilities. Some of these capabilities (e.g., annotating trajectories or adding meta data to the analysis) are unique to *Vision*; other properties are only partially present in other packages (e.g., performing cluster-based, but not cluster-free analysis).

References

[1] van Galen, P. *et al.* Single-cell rna-seq reveals aml hierarchies relevant to disease progression and immunity. *Cell* **176**, 1265 – 1281.e24 (2019).

[2] Subramanian, A. *et al.* Gene set enrichment analysis: A knowledge-based approach for interpreting genome-wide expression profiles. *Proceedings of the National Academy of Sciences* **102**, 15545–15550 (2005).

[3] Kanehisa, M. & Goto, S. Kegg: Kyoto encyclopedia of genes and genomes. *Nucleic Acids Research* **28**, 27–30 (2000).

[4] Ye, C. J. *et al.* Intersection of population variation and autoimmunity genetics in human T cell activation. *Science* **345**, 1254665 (2014).

[5] Abbas, A. R., Wolslegel, K., Seshasayee, D., Modrusan, Z. & Clark, H. F. Deconvolution of blood microarray data identifies cellular activation patterns in systemic lupus erythematosus. *PloS one* **4**, e6098 (2009).

[6] Tusi, B. K. *et al.* Population snapshots predict early haematopoietic and erythroid hierarchies. *Nature* **555**, 54–60 (2018).

[7] Stoeckius, M. *et al.* Simultaneous epitope and transcriptome measurement in single cells. *Nature Biotechnology* **14**, 865–868 (2017).

[8] Kang, H. M., Subramaniam, M. & Targ, S. Multiplexed droplet single-cell RNA-sequencing using natural genetic variation. *Nature Biotechnology* **36**, 89–94 (2017).
