# Supplementary material for: Functional interpretation of single cell similarity maps
Source: Nat Commun. 2019 Sep 26;10:4376. doi: 10.1038/s41467-019-12235-0 (PMC6763499; doi:10.1038/s41467-019-12235-0)
Supplement: Supplementary file 3 — Reporting Summary [file 41467_2019_12235_MOESM3_ESM.pdf]

nature research

Corresponding author(s): Nir Yosef

Last updated by author(s): Jul 31, 2019

Reporting Summary

Nature Research wishes to improve the reproducibility of the work that we publish. This form provides structure for consistency and transparency in reporting. For further information on Nature Research policies, see Authors & Referees and the Editorial Policy Checklist.

Statistics

For all statistical analyses, confirm that the following items are present in the figure legend, table legend, main text, or Methods section.

n/a	Confirmed	
☐	☒	The exact sample size (n) for each experimental group/condition, given as a discrete number and unit of measurement
☐	☒	A statement on whether measurements were taken from distinct samples or whether the same sample was measured repeatedly
☐	☒	The statistical test(s) used AND whether they are one- or two-sided<br>*Only common tests should be described solely by name; describe more complex techniques in the Methods section.*
☐	☒	A description of all covariates tested
☐	☒	A description of any assumptions or corrections, such as tests of normality and adjustment for multiple comparisons
☐	☒	A full description of the statistical parameters including central tendency (e.g. means) or other basic estimates (e.g. regression coefficient) AND variation (e.g. standard deviation) or associated estimates of uncertainty (e.g. confidence intervals)
☐	☒	For null hypothesis testing, the test statistic (e.g. F, t, r) with confidence intervals, effect sizes, degrees of freedom and P value noted<br>*Give P values as exact values whenever suitable.*
☒	☐	For Bayesian analysis, information on the choice of priors and Markov chain Monte Carlo settings
☒	☐	For hierarchical and complex designs, identification of the appropriate level for tests and full reporting of outcomes
☐	☒	Estimates of effect sizes (e.g. Cohen's d, Pearson's r), indicating how they were calculated

Our web collection on statistics for biologists contains articles on many of the points above.

Software and code

Policy information about availability of computer code

Data collection	No software was used in the collection of data.
Data analysis	Data was analyzed using Cell Ranger 2, R 3.5.1 and Python 3.6.6. The analysis tool being presented here is written for R, version 3.4 and higher, and is available at www.github.com/yoseflab/vision

For manuscripts utilizing custom algorithms or software that are central to the research but not yet described in published literature, software must be made available to editors/reviewers. We strongly encourage code deposition in a community repository (e.g. GitHub). See the Nature Research guidelines for submitting code & software for further information.

Data

Policy information about availability of data

All manuscripts must include a data availability statement. This statement should provide the following information, where applicable:

- Accession codes, unique identifiers, or web links for publicly available datasets
- A list of figures that have associated raw data
- A description of any restrictions on data availability

This manuscript uses several public datasets to demonstrate the use of the software tool.

To demonstrate/benchmark the micro-clustering method, published data from Stoeckius et al, 2017, was used and is available in NCBI GEO at accession GSE100866. This data is used in Figure S5.

Published data from Tusi et al, 2018 (available in NCBI GEO at accession GSM2388072) was used in Figure 4 and Figure S4.

Published data from Kang, Subramaniam, and Targ et al, Nature Biotech 2017 (available in NCBI GEO at accession GSE96583) was used in Figures 2, S6 and S7.

Published data from Van Galen et al, Cell 2019 (available in NCBI GEO at accession GSE116256) was used in Figures 3, S1, and S3

Field-specific reporting

Please select the one below that is the best fit for your research. If you are not sure, read the appropriate sections before making your selection.

☒ Life sciences ☐ Behavioural & social sciences ☐ Ecological, evolutionary & environmental sciences

For a reference copy of the document with all sections, see nature.com/documents/nr-reporting-summary-flat.pdf

Life sciences study design

All studies must disclose on these points even when the disclosure is negative.

Sample size	N/A - previously published data was used.
Data exclusions	Data were only excluded based on previously published cell-type labels (e.g. isolating the CD4 T cells), technical labels (removal of 'doublet' marked cells from the Lupus dataset), or standard technical criteria (such as the removal of lowly expressed genes). These criteria are described in the methods of the manuscript.
Replication	N/A - previously published data was used.
Randomization	N/A - previously published data was used.
Blinding	N/A - previously published data was used.

Reporting for specific materials, systems and methods

We require information from authors about some types of materials, experimental systems and methods used in many studies. Here, indicate whether each material, system or method listed is relevant to your study. If you are not sure if a list item applies to your research, read the appropriate section before selecting a response.

Materials & experimental systems

n/a	Involved in the study	
☒	☐	Antibodies
☒	☐	Eukaryotic cell lines
☒	☐	Palaeontology
☒	☐	Animals and other organisms
☒	☐	Human research participants
☒	☐	Clinical data

Methods

n/a	Involved in the study	
☒	☐	ChIP-seq
☒	☐	Flow cytometry
☒	☐	MRI-based neuroimaging
